# Surface Layer Protein Pattern of *Levilactobacillus brevis* Strains Investigated by Proteomics

**DOI:** 10.3390/nu14183679

**Published:** 2022-09-06

**Authors:** Maria Fiorella Mazzeo, Anna Reale, Tiziana Di Renzo, Rosa Anna Siciliano

**Affiliations:** Institute of Food Sciences, National Research Council (CNR-ISA), Via Roma 64, 83100 Avellino, Italy

**Keywords:** *L. brevis*, proteomics, probiotics, surface layer proteins, surface associated proteins, lactic acid bacteria

## Abstract

The outermost constituent of many bacterial cells is represented by an S-layer, i.e., a semiporous lattice-like layer composed of self-assembling protein subunits called S-layer proteins (Slps). These proteins are involved in several processes, such as protecting against environmental stresses, mediating bacterial adhesion to host cells, and modulating gut immune response. Slps may also act as a scaffold for the external display of additional cell surface proteins also named S-layer associated proteins (SLAPs). *Levilactobacillus brevis* is an S-layer forming lactic acid bacterium present in many different environments, such as sourdough, milk, cheese, and the intestinal tract of humans and animals. This microorganism exhibits probiotic features including the inhibition of bacterial infection and the improvement of human immune function. The potential role of Slps in its probiotic and biotechnological features was documented. A shotgun proteomic approach was applied to identify in a single experiment both the Slps and the SLAPs pattern of five different *L. brevis* strains isolated from traditional sourdoughs of the Southern Italian region. This study reveals that these closely related strains expressed a specific pattern of surface proteins, possibly affecting their peculiar properties.

## 1. Introduction

Probiotics are microorganisms present in the intestinal tract and in many fermented foods that positively affect human health by aiding digestion and absorption of dietary nutrients, strengthening intestinal barrier function, modulating immune response, and enhancing antagonism toward pathogens [1]. Cell surface proteins constitute the first-line of contact between bacteria and the environment and/or host, and are directly involved in several molecular mechanisms responsible for beneficial health effects [2]. In several probiotic bacteria, including those belonging to the genus *Lactobacillus*, the outermost constituent of the cell wall is often represented by an S-layer, which is a semiporous, symmetric, crystalline lattice-like layer composed of self-assembling protein subunits called S-layer proteins (Slps) [3,4]. The subunits, held together and attached to cell wall carbohydrates by non-covalent interactions, can account for up to 15% of total bacterial proteins. The Slps of lactobacilli are characterized by a smaller size (25–71 kDa) and a higher predicted pI value (9.4–10.4) compared to those of other bacteria [5].

Slps are involved in several processes, including maintaining cell shape, acting as molecular sieves, serving as binding sites, protecting against environmental stresses, which are essential functions for ensuring survival and viability of probiotics in the gastrointestinal trait (GIT). In addition, they have a key role in mediating bacterial adhesion to host cells and modulating the gut immune response [4,6]. The S-layer could also protect cells during technological processes such as freeze-drying and anchor proteolytic enzymes to the cell surface, thus providing important advantages to commercial starter strains [7]. Moreover, Slps may act as a scaffold for the external display of additional cell surface proteins, referred to as S-layer associated proteins (SLAPs). These proteins exhibit a wide array of biological functions, encompassing protein and carbohydrate degradation, cell wall organization and cell shape regulation, host adhesion, and immunomodulation. SLAPs are non-covalently anchored to the cell wall and easily extracted from intact bacteria by the action of chaotropic agents, such as lithium chloride and guanidine chloride [8,9].

In the last decades, proteomics has played a key role in the identification of surface exposed proteins and the investigation of the dynamic molecular crosstalk between probiotics and host [10]. In the pioneering study carried out by Johnson and colleagues [8], an ad-hoc protocol for the extraction of SLAPs was set up and 37 SLAPs were identified for the first time in *Lactobacillus acidophilus* NCFM. More recently, due to improvements in proteomic methodologies and instrumentation, Klotz and colleagues [11] obtained a more comprehensive picture of the non-covalent exoproteome of *L. acidophilus*, identifying as many as 352 SLAPs and highlighting that the relative abundance of most SLAPS was actually modulated by growth phases (logarithmic vs. early stationary growth phases). Proteomics has also been fundamental to discover the presence of some cytoplasmic housekeeping proteins (metabolic enzymes, molecular chaperones, translational elongation factors, ribosomal proteins, etc.) in the SLAPs assortment. Such proteins, defined “moonlighting proteins”, display diverse biological functions in different cellular locations and lack any extra-cytoplasmic sorting sequence or binding domain, thus non-canonical secretion pathways have been hypothesized. Moonlighting proteins are generally involved in adhesion to host epithelia and extracellular matrices, as well as in the modulation of the immune response [12].

*Levilactobacillus brevis* is a lactic acid bacterium (LAB) that produces Slps. This microorganism is present in many different environments, such as sauerkraut, sourdough, silage, milk, cheese, mouth and intestinal tract of humans and animals, and is involved in the production of a wide spectrum of fermented products [13,14,15,16,17]. Interestingly, the potential involvement of Slps in its probiotic and biotechnological features was documented [7,18,19,20,21,22,23]. As for other Lactobacilli, multiple S-layer protein genes were reported in *L. brevis* genomes. In fact, three genes coded for Slps in the genomes of *L. brevis* UCCLB556 [13], *L. brevis* SF9B [22], and TMW 1.2112 [24]. The expression of S-layer protein genes is strain specific and can be modulated by growth and/or stress conditions [25,26].

Slps synthetized by *L. brevis* show some peculiar structural and functional features. In fact, the N-terminal regions of Slp A, B, C, and D have a high sequence similarity, whereas the C-terminal regions are quite dissimilar [5,25]. The C-terminal domain of SlpA is actually involved in the self-assembly process of the protein subunits into a regular S-layer array and the N-terminal region exhibits a high-predicted pI, and comprises the cell wall binding domain. These domains are located in a reverse order compared to that of all other *Lactobacillus* Slps. Moreover, *L. brevis* Slps apparently interact with non-teichoic acid polysaccharides (neutral polysaccharides), while teichoic acids represent the cell wall receptors of Slps of the *L. acidophilus* group [5,26].

The aim of the present study was to depict the Slps pattern of five different *L. brevis* strains isolated from traditional sourdoughs typical of the Southern Italian region, biotypized by RAPD-PCR (Randomly Amplified Polymorphic DNA Polymerase Chain Reaction) and to catalogue the S-layer associated proteome of these strains.

## 2. Materials and Methods

### 2.1. Bacterial Strains and Growth Conditions

Five strains of *Levilactibacillus brevis* were used in this study. In detail, *L. brevis* PA6 [27], *L. brevis* A4, A7, and M4 [28] were previously isolated from traditional wheat sourdoughs and stored in the culture collection of the Institute of Food Sciences, National Research Council, Avellino, Italy. The strain *L. brevis* DSMZ 20054 (, named TS in this work) was obtained from DSMZ-German Collection of Microorganisms and Cell Cultures GmbH (Braunschweig, Germany) and used as reference strain. The strains were maintained as frozen stock at −80 °C in reconstituted 11% (*w*/*v*) skim milk (Thermo Fisher Scientific, Waltham, MA, USA) containing 0.1% (*w*/*v*) of ascorbic acid. Before experimental use, *L. brevis* strains were subcultured twice in MRS broth (Thermo Fisher Scientific), pH 6.8, for 16 h at 28 °C (exponential growth phase).

### 2.2. DNA Extraction and Biotypization of Strains

RAPD-PCR analysis was carried out for the differentiation of closely related strains of *L. brevis*. DNA extraction and biotypization of strains were performed as described by Reale et al. [29]. Cell pellet was subjected to DNA extraction according to Querol et al. [30] with minor modifications. Protocols for DNA extraction and amplification and for cluster analysis of electrophoresis band profiles are reported in Table S1 [31,32].

### 2.3. Extraction and Digestion of Surface Proteins for Proteomic Analyses

Bacterial cells (100 mL) were cultivated (1% *v*/*v*, inoculum) in Man, Rogosa and Sharpe broth (MRS) (Thermo Fisher Scientific) (pH 6.8) at 28 °C for approximately 16 h (exponential growth phase). Cells were harvested by centrifugation (10,000× *g*, 10 min, 4 °C) and washed twice with 50 mmol/L Tris-HCl pH 7.5.

Surface proteins were extracted by using the LiCl protocol of Lortal and colleagues [33] with minor modifications. Protein samples were digested with Sequencing Grade Modified Trypsin (Promega, Madison, WI, USA). Protocols for surface protein extraction and tryptic digestion are reported in detail in Table S2.

### 2.4. LC-MS/MS Analysis, Protein Identification, and Label-Free Quantification (LFQ) Analyses

Tryptic peptide mixtures were submitted to LC-MS/MS analysis using a Q-Exactive^TM^ mass spectrometer (Thermo Fisher Scientific) interfaced with an UltiMate 3000 RSLCnano LC system (Thermo Fisher Scientific).

Two biological replicates were analyzed for each bacterial strain and three technical replicates were acquired for each biological replicate.

Protein identification and quantification were achieved by using the MaxQuant software (version 1.6.3.4) (developed by the Computational Systems Biochemistry, The Max-Planck-Institute for Biochemistry, Martinsried, Germany) [34]. The Perseus software (version 1.6.0.7)) (developed by the Computational Systems Biochemistry, The Max-Planck-Institute for Biochemistry, Martinsried, Germany) was used for further processing the label free quantification data obtained by MaxQuant analysis and to build up the heat maps reporting the LFQ values for each protein in the six replicates of each strain [35].

The putative relative abundance level of each Slp in a single strain was calculated using a spectral counting approach [36].

Detailed protocols for LC-MS/MS data acquisition and parameters used for data processing are reported in Table S3.

### 2.5. Bioinformatics and Functional Analyses

The Protein Basic Local Alignment Search Tool (Protein BLAST) was used to compare protein sequences of identified uncharacterized proteins to protein sequences present in the database UniProtKb (Restrict by taxonomy: *Levilactobacillis brevis* (1580); https://blast.ncbi.nlm.nih.gov, accessed on 20 April 2022). Identified proteins were analyzed using the SignalP 5.0 server (http://www.cbs.dtu.dk/services/ SignalP, accessed on 5 May 2022), which predicts the presence and location of signal peptide cleavage sites in amino acid sequences for translocation across cell membranes [37]. Functional classification of SLAPs was retrieved from The Gene Ontology (GO) knowledgebase [38]. The MoonProt database (http://www.moonlightingproteins.org/, accessed on 5 May 2022) was used to retrieve putative moonlighting features of the identified proteins [39]. The Venn diagram was built using the web-based tool InteractiVenn (http://www.interactivenn.net/ accessed on 5 May 2022) [40].

**Figure 1:**
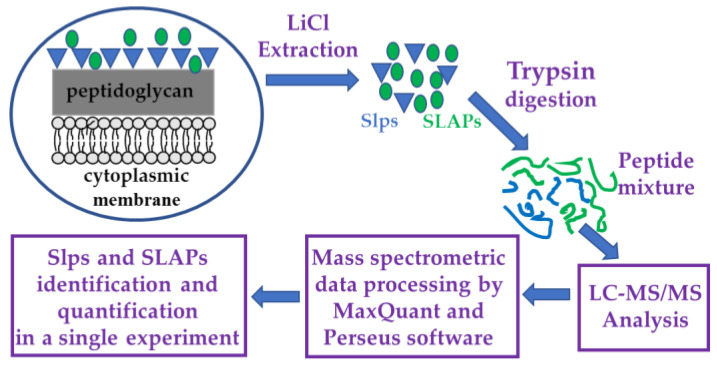
Flow chart of the experimental design. Surface layer proteins (Slps) are indicated with blue triangles and surface layer associated proteins (SLAPs) with green circles.

The experimental design is outlined in Figure 1.

## 3. Results and Discussion

A quantitative proteomic study was designed to identify surface layer proteins (Slps) and surface layer associated proteins (SLAPs) of five different *L. brevis* strains. To the best of our knowledge, such a rapid and straightforward analytical approach that does not require any targeted extraction of SLAPs is here reported for the first time. The patterns of Slps and SLAPs of *L. brevis* were characterized for the first time and these data could give important clues on functional and technological properties of these strains. Furthermore, RAPD-PCR analysis was used for determining genetic fingerprinting of the five strains of *L. brevis* for the differentiation of closely related strains.

### 3.1. Proteomic Analysis of Surface Layer Proteins (Slps) of L. brevis Strains

Shotgun proteomics provided a detailed picture of the Slps expressed by the five *L. brevis* strains. Ten different Slps were identified, and in particular, beyond the already well-characterized Slps A, B, C, and D, we identified the SlpM, which shared a high sequence identity (93.6%) with SlpB and three S-layer proteins (A0A856KME7, A0A1W6NKR1, A0A1W6N798), which had more than 50% sequence identity with SlpA (Table 1, Table 2 and Table S4 (sheet 1)). In addition, BLAST analysis revealed that the uncharacterized protein with accession A0A1W6NJ64 shared a high sequence identity (88.5%) with SlpC and the uncharacterized protein A0A856KHL8 shared a high sequence identity (97.6%) with the S-layer protein A0A1W6N798 and 61.5% sequence identity with SlpA. Therefore, these two proteins could represent additional Slps of *L. brevis* (Table 2). As expected, an N-terminal signal peptide was present in all these proteins that allowed translocation across cell membranes, as assessed using the Signal P software (Table S4 (sheet 1)).

The Slp A, B, and C and the S-layer protein A0A1W6N798 were expressed, although in different amounts, by all the analyzed strains, while four strains, except M4, produced SlpD. SlpM, Slps A0A1W6NJ64, and A0A856KME7 were specifically identified in just one strain (the first two in A4, and the last one in A7). Interestingly, this study reports for the first time, evidence of the expression of ten Slps of *L. brevis* and the simultaneous presence of up to eight different Slps on the S layer in a single strain (A7 strain).

**Figure 2:**
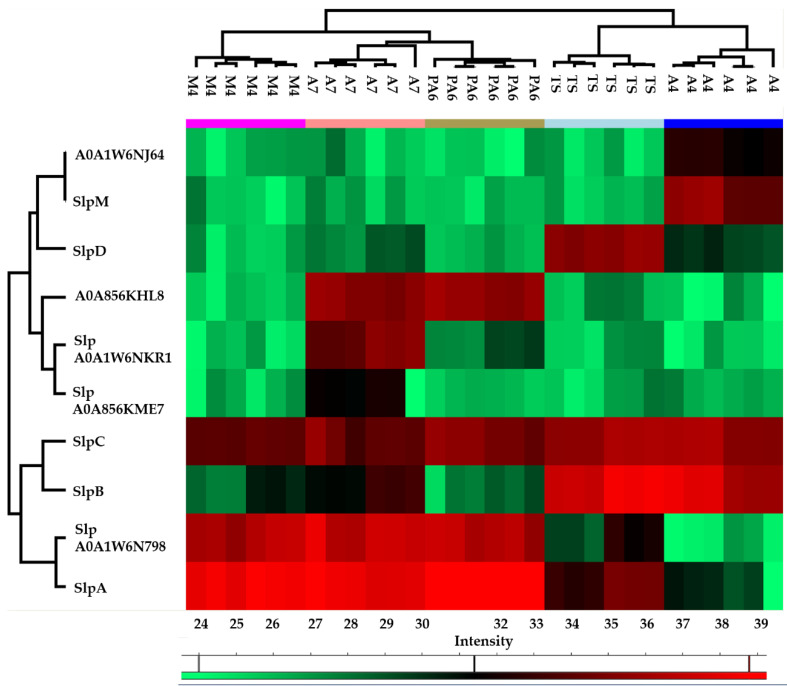
Heatmap obtained reporting protein abundance (LFQ values) of each surface layer protein (Slp) in technical and biological replicates of each strain. The green and red color ranges refer to the lower and higher abundance, respectively (heat maps were obtained by Perseus).

The heat map obtained from the quantitative proteomic data showed that the five strains were grouped in two clusters: the first one included PA6, A7, and M4, and was characterized by a higher abundance of SlpA and S-layer protein A0A1W6N798 while the second one included A4 and TS, and was characterized by a higher abundance of SlpB and SlpC (Figure 2).

**Figure 3:**
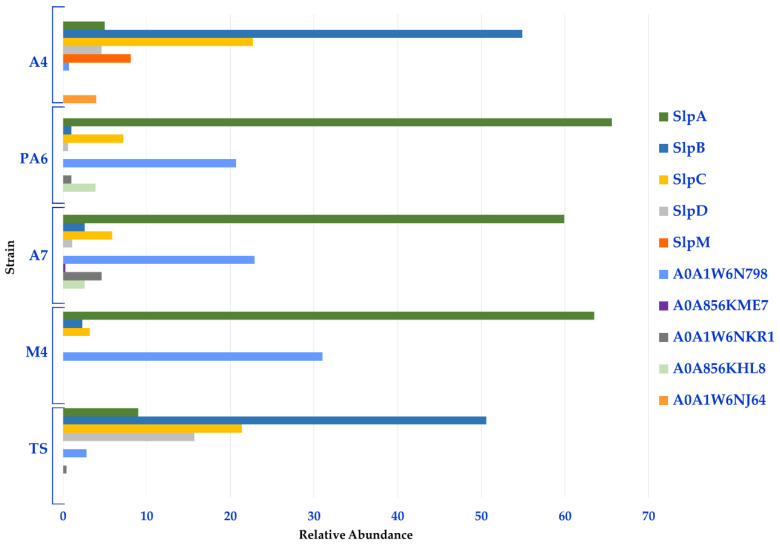
Relative distribution of the Slps identified in each *L. brevis* strain.

Moreover, a semi-quantitative analysis based on the spectral counting method (MS/MS counts) was performed to gather information on the relative distribution of the Slps identified in each strain. In line with the heat map, this analysis revealed that Slp A, B, C, D, and A0A1W6N798 constituted the Slps core of these strains; in fact, SlpA accounted for about 60% of the Slps present on the surface of PA6/A7/M4 while the related protein A0A1W6N798 accounted for more than 20%. Therefore, the S-layer of PA6, A7, and M4 strains was formed for more than 90% by SlpA and proteins with high identity with SlpA. SlpB and SplC were also expressed by these strains and accounted for about 10%. On the other hand, SlpB and SlpC accounted for about 50% and 20% of the S-layer of A4 and TS strains, respectively. SlpD was mainly present in TS (16%) and in a minor amount in A4 (5%), while SlpM was specifically expressed by A4 (8%). For this group of strains, SlpB, SlpC, SlpD, and SlpM accounted for about 90% of Slps while SlpA was present in a minor amount (5% and 9% in A4 and TS, respectively) (Figure 3, Table S4 (sheet 2)).

These findings paralleled with data obtained by the label free quantitative proteomic analysis performed on the main Slps by comparing the LFQ value of each Slp in A4, PA6, A7, and M4 strains with the corresponding LFQ value in TS (considered as the reference strain). In fact, in PA6/A7/M4 strains, SlpA was highly abundant while SlpB and SlpD were more abundant in TS. In A4, SlpB and SlpC showed no differences in abundance level compared to TS, while SlpA and SlpD were present in lower amount (Table 3 and Table S4 (sheet 3)).

Previous studies had already reported the ability of *L brevis* ATCC 14869 (corresponding to strain DSMZ 20054 and referred to as TS in this work) to produce both SlpB and SlpD under aerobic growth conditions and synthesize only SlpB under anaerobic conditions, while the *splC* gene was considered a silent gene [25]. Remarkably, our proteomic analysis highlighted for the first time that TS not only expressed SlpB and D but also a significant amount of SlpC.

On the other hand, the strains PA6/A7/M4 of *L. brevis* mainly expressed SlpA (about 90% relative abundance). Interestingly, the S-layer of *L. brevis* ATCC 8287 is also formed by SlpA [41], which binds with high affinity to fibronectin and laminin, thus mediating the adhesion process to host cells [26,42]. Adhesion ability is a strain specific feature and can be deeply affected by the expressed Slps pattens; in fact, *L. brevis* 14,869, mainly producing SlpB and SlpD, exhibited a greater adhesion to human enterocytes in vitro compared to *L. brevis* ATCC 8287 [26]. Moreover, recent studies highlighted that the Slps expressed by *L. brevis* ATCC 14869 are directly involved in the inhibition of bacterial infection and modulation of DC cytokine production [43,44].

Our results showed that the analyzed strains produced a more complex Slps pattern compared to the ones reported so far, which could possibly lead to differences in the functional properties of the two groups of strains (A4/TS vs. PA6/A7/M4).

### 3.2. Proteomic Analysis of Surface Layer Associated Proteins (SLAPS) of L. brevis Strains

**Figure 4:**
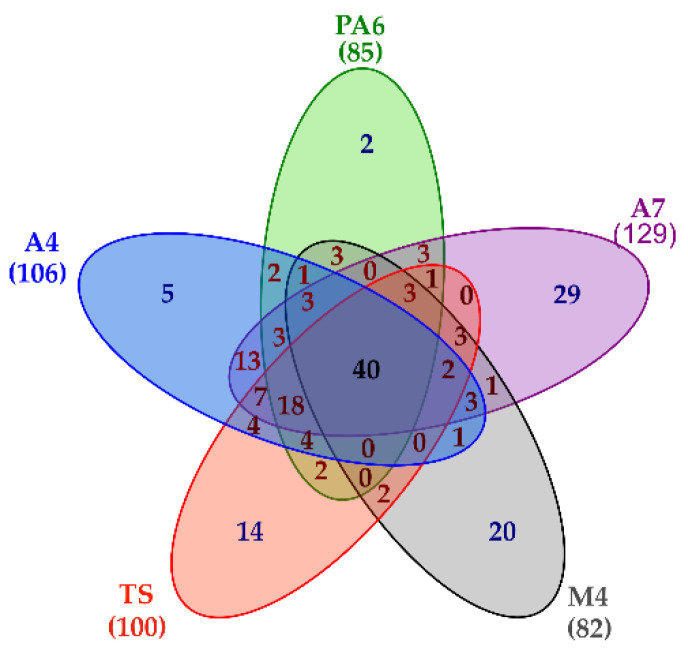
Venn diagram summarizing proteomic results. Lists including the accession of proteins reliably identified in each strain were used to build the diagram. The number of surface layer associated proteins (SLAPs) present in each strain is reported in bracket.

Besides providing a detailed picture of the *L. brevis* Slps pattern, the shotgun proteomic approach also led to the simultaneous identification of 189 SLAPs present at least in one of the five strains. In particular, 106 proteins were reliably identified in A4, 85 proteins in PA6, 129 proteins in A7, 82 proteins in M4, and 100 proteins in TS. A common core of 40 proteins was present on the surface of all the strains, although in different amounts, while 70 proteins were specifically present in only one strain (Figure 4, Table S5 (sheet 1)).

Bioinformatics processing of the protein sequences included in this dataset using the SignalP tool highlighted that 90 proteins (48%) were predicted to contain a signal peptide for translocation across cell membrane. In addition, we also identified an acetyltransferase and the cell surface protein A0A7Z6MNN6 containing a transmembrane helix, and three cell membrane proteins (ATP synthase subunit alpha, ATP synthase subunit beta, and high-affinity heme uptake system protein IsdE) (Table S6).

According to Gene Ontology annotations, most of the predicted extracellular proteins had the following molecular function: metalloendopeptidase, carboxypeptidase, N-acetylmuramoyl-L-alanine amidase, and hydrolase activities. Unfortunately, no functional annotation could be retrieved for 29 proteins with the N-terminal signal peptide and annotated as uncharacterized proteins. In fact, no significant identity with proteins having a characterized biological function was retrieved by BLAST analysis for these proteins.

As to the biological function annotations, many of the extracellular proteins were involved in peptidoglycan biosynthetic and catabolic processes that then affect cell wall organization and regulation of cell shape. In particular, D-alanyl-D-alanine carboxypeptidase, penicillin-binding proteins, L,D-transpeptidase, putative ErfK/YbiS/YcfS/YnhG family protein are part of the biosynthetic machinery, while N-acetylmuramoyl-L-alanine amidase and 1,4-beta-N-acetylmuramidase are reported to participate to the peptidoglycan degradation (Table S6). These proteins, together with muramidase and peptidoglycan endopeptidase, are endogenous enzymes capable of cleaving covalent bonds in polymeric peptidoglycan and/or in its soluble fragments and participate in bacterial cell wall growth and its regulation in different lysis phenomena [45].

Proteomic analysis also led to the identification of 95 proteins with cytoplasmic subcellular localization. However, their presence on the cell surface cannot be ruled out. In fact, twenty of these proteins were annotated in the MoonProt database (http://www.moonlightingproteins.org/ accessed on 5 May 2022) as bacterial moonlighting proteins [39] (Table S6). In particular, the localization of enolase, glyceraldehyde 3-P-dehydrogenase, elongation factor Tu, 60 kDa chaperonin (GroEL), chaperone protein DnaK (HSP70), and pyruvate kinase on the cell surface of lactic acid bacteria has already been reported. These proteins could act as mucin, plasminogen, fibronectin, and laminin binding proteins, thus being involved in adhesion processes (http://www.moonlightingproteins.org/ accessed on 5 May 2022).

As for the presence of other cytoplasmic proteins in the identified SLAPs, it should be taken into consideration that minor contamination of these proteins could be due to a partial cell lysis that occurs during cell growth or sample preparation. Interestingly, nine proteins identified in this study (2,3-bisphosphoglycerate-dependent phosphoglycerate mutase, 30S ribosomal protein S2, elongation factor Tu (EF-Tu), enolase, glyceraldehyde-3-phosphate dehydrogenase, penicillin-binding protein, pyruvate kinase, serine protease, trigger factor) have already been reported among the 25 most abundant SLAPs of *L. acidophilus* in the logarithmic phase in the study carried out by Klotz and co-workers [11].

The heat map obtained by considering the LFQ values for each SLAP in the different strains showed that the M4 strain is the most peculiar one while the other strains had a more similar pattern of SLAPs. The lower part of heat map included a cluster of 22 proteins highly abundant in all the strains; interestingly, among those, we found four proteins with N-acetylmuramoyl-L-alanine amidase activity and the 1,4-beta-N-acetylmuramidase, which are involved in peptidoglycan catabolic process (Figure S1, Table S5 (sheet 3)).

Proteomic data highlighted that the analyzed *L. brevis* strains showed peculiar features of the SLAPs pattern. In fact, 29, 20, and 14 proteins were reliably identified only in A7, M4, and TS, respectively. On the other hand, fewer proteins were specifically identified only in A4 or PA6 (five and two proteins, respectively). Interestingly proteins belonging to the ATP binding cassette (ABC) transporter complex that are involved in transmembrane transport were specifically present on the cell surface of the A7 strain (Table S5).

In addition, label-free quantitative analysis performed considering TS as a reference strain, showed that 17 proteins were differentially abundant in A4 (eight and nine proteins had higher and lower abundance, respectively); 16 proteins were differentially abundant in PA6 (12 and 4 proteins had higher and lower abundance, respectively), 32 proteins were differentially abundant in A7 (11 and 21 proteins had higher and lower abundance, respectively), and 30 proteins were differentially abundant in M4 (12 and 18 proteins had higher and lower abundance, respectively). In all the strains, two proteins (D-alanyl-D-alanine carboxypeptidase dacA and Fucose-binding lectin II) were present in higher abundance than in TS. A4, A7, and M4 strains showed the same trend of abundance for five proteins (cell surface protein and muramidase were present in higher abundance, whereas 50S ribosomal protein L11, 50S ribosomal protein L5, and enolase were present in lower abundance). In addition, A7 and M4 showed the same trend of abundance for six proteins (two proteins had higher abundance (Big_6 domain-containing protein and Histidine phosphatase family protein), and four proteins had lower abundance (50S ribosomal protein L15, DNA-binding protein HU, 50S ribosomal protein L27, Putative lipoprotein, respectively). The abundance of five proteins was specifically modulated in A4, five in PA6, 11 in A7, and 13 in M4.

Differences in SLAPs pattern could affect the probiotic and technological properties of these strains.

### 3.3. Biotypization of Levilactobacillus brevis Strains

**Figure 5:**
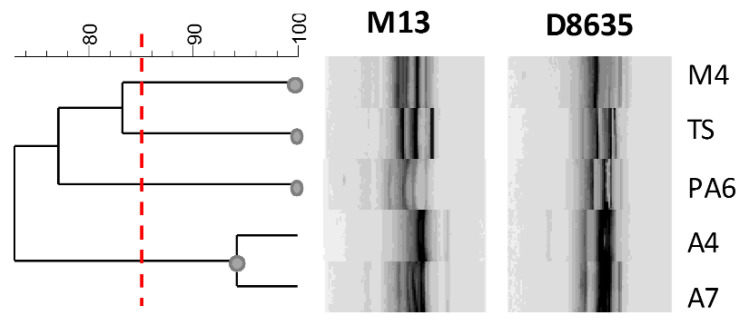
Dendrogram showing the similarity among RAPD-PCR profiles of *L. brevis* strains. The red line indicates the cluster cut-off value of 85%, which delimitates the main clusters.

In this study, RAPD-PCR analysis was used to detect intraspecific differences among *L. brevis* strains. The fingerprintings are reported in Figure 5. The primers used a generated number of amplified DNA fragments ranging from 3 to 10 amplicons and the size of amplified fragments ranging from 200 to 2000 bp.

Profiles obtained with RAPD-PCR highlighted a sensible biodiversity among the analyzed strains. Considering genetic relationships between strains with more than 85% of similarity of the RAPD genotypes, four clusters were distinguished (Figure 5). Although the strains were isolated from the same food matrix, they showed a sensible genetic biodiversity with the exception of the strains A4 and A7. Interestingly, these last strains with a similar fingerprinting profile presented very different Slps and SLAPs profiles, confirming that the phenotypic and intrinsic characteristics are specific of the strains and that characterization of surface proteins could contribute to the estimation of their functional and technological features.

## 4. Conclusions

The S-layer proteins of five *L. brevis* strains isolated from traditional sourdoughs were characterized, showing that these closely related strains actually exhibited a very specific Slps pattern, including up to eight different protein forms.

The role of Slps in the probiotic properties of several bacteria was documented, thus suggesting a possible exploitation of their potential as paraprobiotics and/or post-biotics. As a matter of fact, the high abundance level of these proteins in the bacterial cells (up to 15% total protein content) and the possibility to easily isolate these components, make Slps attractive candidates for the design of new drugs or functional foods enriched with such biomolecules. However, the biosynthesis of the strain specific pattern of Slps cannot be overlooked, and, in this perspective, proteomics could be fundamental to investigate Slps diversity, thus contributing to select bacterial strains tailored to fulfill specific health benefits.

## Figures and Tables

**Table 1:** Proteomic analysis of the surface layer protein (Slps) pattern in the five *L. brevis* strains.

Accession	Protein Name	Gene Name	Seq. Cov. [%]	Mol. Weight [kDa]	pI	A4 ^1^	PA6 ^1^	A7 ^1^	M4 ^1^	TS ^1^	n. of Strains
G1UE81	Surface layer protein A	SlpA	66.1	48.17	9.54	x	x	x	x	x	5
Q8GFE5	Surface layer protein B	slpB	92.1	50.92	9.54	x	x	x	x	x	5
Q8GFE4	Surface layer protein C	slpC	84.6	48.86	9.66	x	x	x	x	x	5
Q8GFE3	Surface layer protein D	SlpD	77.5	44.58	9.66	x	x	x		x	4
H9BQS1	Surface layer protein M	slpM	63.3	51.46	9.54	x					1
A0A1W6N798	S-layer protein	AZI09_01470	84.3	47.76	9.60	x	x	x	x	x	5
A0A856KME7	S-layer protein	UCCLB556_pD0019	13.8	47.89	9.68			x			1
A0A1W6NKR1	S-layer protein	AZI11_14340	27.8	49.17	9.48		x	x		x	3
A0A856KHL8	Uncharacterized protein	UCCLB556_2008	75.3	47.68	9.56		x	x			2
A0A1W6NJ64	Uncharacterized protein	AZI11_11480	55.2	48.42	9.65	x					1

^1^ Proteins reliably identified in the strain.

**Table 2:** BLAST analysis of the Slps identified in the five *L. brevis* strains.

Query	Protein Name	Sequence Identity (%)	Similar Protein Accession	Protein Name
H9BQS1	Surface layer protein SlpM	93.6	Q8GFE5	Surface layer protein SlpB
A0A856KME7	S-layer protein	57.7	G1UE81	Surface layer protein A
A0A1W6NKR1	S-layer protein	58.0	G1UE81	Surface layer protein A
A0A1W6N798	S-layer protein	60.6	G1UE81	Surface layer protein A
A0A1W6NJ64	Uncharacterized protein	88.5	Q8GFE4	Surface layer protein SlpC
A0A856KHL8	Uncharacterized protein	61.5	G1UE81	Surface layer protein A

**Table 3:** Label free quantitative proteomic analysis of the main identified Slps in the five *L. brevis* strains.

Accession	Protein Name	Gene Names	A4 vs. TS Fold Change	A6 vs. TS Fold Change	A7 vs. TS Fold Change	M4 vs. TS Fold Change
G1UE81	SlpA	slpA	−4.76	5.20	4.41	4.62
Q8GFE5	SlpB	slpB	NDA	−9.98	−5.67	−8.57
Q8GFE4	SlpC	slpC	NDA	NDA	−1.56	−1.83
Q8GFE3	SlpD	SlpD	−5.77	−9.30	−7.24	nd
A0A1W6N798	Slp A0A1W6N798	AZI09_01470	−5.44	5.95	6.52	5.82

TS is the reference strain; NDA: Not differentially abundant, nd: not reliably detected. Protein fold changes were calculated as the difference of log_2_ of mean protein LFQ values in A4, PA6, A7, or M4 and log_2_ of mean protein LFQ in TS.

## Data Availability

Data presented in this study are available on request from the corresponding author.

## Supplementary Materials

The following supporting information can be downloaded at: https://www.mdpi.com/article/10.3390/nu14183679/s1, Figure S1: Heatmap obtained reporting protein abundance (LFQ values) of each SLAP in technical and biological replicates of each strain. The green and red color ranges refer to the lower and higher abundances, respectively (heat maps were obtained by Perseus). Table S1: DNA extraction, primer, and amplification conditions for RAPD-PCR, cluster analysis of band profiles. Table S2: Detailed protocols for extraction and digestion of surface proteins. Table S3: Detailed protocols for LC-MS/MS, Protein Identification and Label-Free Quantification (LFQ) Analyses. Table S4: Identification of Slps in the five *L. brevis* strains (sheet 1); Relative abundance level of each Slp in a single strain calculated using a spectral counting approach (sheet 2); Label free quantitative proteomic analysis performed comparing the LFQ value of each Slp in A4, PA6, A7, M4 to the corresponding LFQ value in TS (considered the reference strain) (sheet 3). Table S5: Identification of SLAPs in the five *L. brevis* strains (sheet 1); Label free quantitative proteomic analysis performed comparing the LFQ value of each SLAP in A4, PA6, A7, M4 to the corresponding LFQ value in TS (considered the reference strain) (sheet 2); List of 22 proteins highly abundant in all the strains (sheet 3); Lists of proteins reliably detected in a single strain (sheet 4–8). Table S6: Cell location and Functional classification of the identified SLAPs.
