# Supplementary material for: Surface Layer Protein Pattern of Levilactobacillus brevis Strains Investigated by Proteomics
Source: Nutrients. 2022 Sep 6;14(18):3679. doi: 10.3390/nu14183679 (PMC9504196; doi:10.3390/nu14183679)
Supplement: Supplementary file 1 [file nutrients-14-03679-s001.zip › Figure S1.pdf]

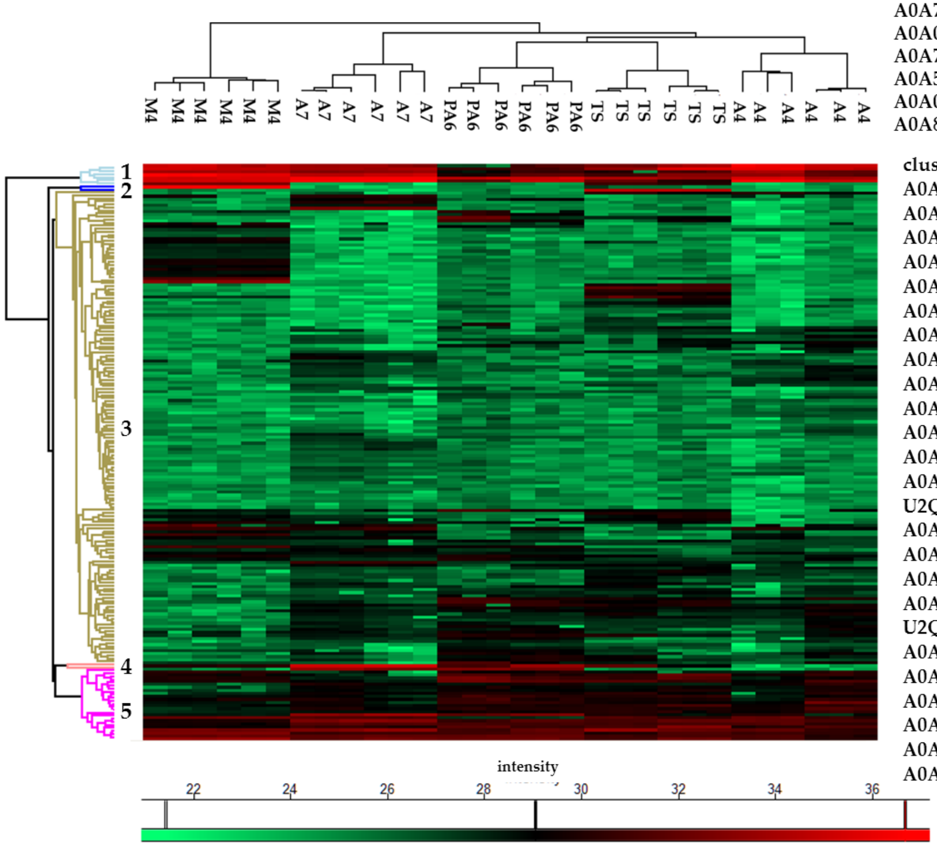

cluster 1
A0A7Z6MKY8
A0A0C1M466
A0A7Z6MNM2
A0A5B7XZ35
A0A0D0G3E1
A0A856KH62

cluster 2
A0A0C1Q7L1
A0A2Z3RA58

cluster 4
A0A7Z6MQ45
A0A7Z6QJH9

cluster 3
A0A0C1M2C5
A0A3B8EQE4
A0A7Z6MN88
A0A1W6N7E5
A0A856KK47
A0A1W6N6U3
A0A3B8ECC2
A0A3B8EB97
A0A7Z6MPS7
A0A2Z3REC8
A0A0C1M8Y4
A0A7Z6QHR0
A0A7Z6QIS1
U2QJY4
A0A1W6N6T1
A0A378HPA1
A0A1W6N6J9
A0A0C1Q7S8
U2QQE4
A0A1W6N6W7
A0A378HJ74
A0A1W6NAL1
A0A0C1PUB4
A0A2Z3R7E4
A0A2Z3RCR9
A0A378HI03
A0A378HD43
A0A1W6N8N0
A0A378HKL0
A0A378HHX5
A0A0C1M6V3
A0A0C1PUT1
A0A378HDI2
U2PCM2
A0A0C1PT31
A0A0C1PPN7
A0A0C1M121
A0A0C1LXH1
A0A2Z3RCA7
A0A5B7XWW9
A0A378HI62
A0A5B7Y4W3
A0A2A3U0S5
A0A3B8F4T7
A0A0C1LXH6
A0A0C1PV79
A0A2A3TT68
A0A0C1LY44
A0A1X0XPF0
A0A5B7Y1Q7
A0A5B7XYC9
A0A1W6NBY1
A0A0C1PNE4
A0A0C1PYK8
A0A0C1M6I4
A0A7Z6QJB4
A0A2A3TWJ8
A0A0C1LXI0
A0A0C1M5H0
A0A0C1PXK9
A0A0C1PL57
A0A7Z6MKX8
A0A1T1C653
A0A0C1M885
A0A2A3TUS7
A0A0C1LXH7
A0A0C1PVD6
A0A1W6N9K8
A0A7Z6QIV8
A0A856KJK1
A0A1W6NDZ2
A0A0C1PUH0
A0A1W6NEE8
A0A7Z6MPF8
A0A5B7Y2D7
A0A7Z6MR87
A0A0C1Q7Y1
A0A7Z6QKE5
A0A1W6NB42
A0A0D0G7P1
A0A0D0GUH1
A0A7Z6MMI0
A0A1W6N8K2
A0A7Z6MLM3
A0A0C1Q4I4
A0A0C1PSU0
A0A7Z6QIM1
A0A0C1Q4N7
A0A7Z6QJP7
A0A856KDA4
A0A2A3TWY4
A0A7Z6QKN1
A0A2Z3RAY3
A0A7Z6MQR5
A0A7Z6MN77
A0A856KJD2
A0A7Z6MMB1
A0A7Z6MNQ0
A0A7Z6MLW8
A0A7Z6MNT3
A0A7Z6QJJ5
A0A1X0XJU5
A0A5B7XYQ3
A0A856KI39
A0A0C1Q0J6
A0A0C1M9B2
A0A5B7XXB2
A0A5B7Y305
A0A0C1M988
A0A1W6NI80
A0A7Z6QN82
A0A378HPP8
A0A7Z6MP80
A0A2Z3RA17
A0A1X0XML3
A0A2Z3R5T4
A0A2A3U0V4
A0A7Z6MM31
A0A378HEA5
A0A856KLQ3
A0A2Z3R9E3
A0A2Z3R8J5
A0A7Z6MQJ9
A0A0C1PL61
Q93TM6
A0A0C1Q5X8
A0A0C1PTT3
A0A7Z6MNN6
A0A7Z6MLB6
A0A0C1PNC9
A0A0C1Q0K6
A0A0C1LY48
A0A0C1M5G7
A0A0C1PUK2
A0A7Z6MPS2
A0A1X0XVA6
A0A2Z3RC35
A0A0C1PND9
A0A0C1PND4
A0A0C1PYD3
A0A3B8ER12
A0A0C1PNB2
A0A0C1M617
A0A7Z6QML4
A0A1W6NAR4
A0A0C1PL67
A0A0C1PW29
A0A0C1Q7C7
A0A2Z3R9T1
A0A0C1PL48
A0A5B7XZH6
A0A1W6N9F0
A0A3B8EE47
A0A5B7XZ02
A0A1X0XU92

cluster 5
A0A3B8ERW9
A0A0C1PW84
A0A0C1Q0L0
A0A0C1PL63
A0A0C1MBZ5
A0A0C1MDP1
A0A0C1M5H4
A0A7Z6QHX9
A0A3B8EXN5
A0A0C1LXG8
A0A0C1PUP3
A0A0D0G6W0
A0A0C1LXH3
A0A0C1M5F8
A0A7Z6QK90
A0A2A3TZM2
A0A0C1PSK3
A0A7Z6MLA7
A0A0C1QAC7
A0A7Z6MLH5
A0A7Z6QND1
A0A1W6NH69
A0A1W6NKA8

Figure S1: Heatmap obtained reporting protein abundance (LFQ values) of each SLAP in technical and biological replicates of each strain. The green and red color ranges refer to the lower and higher abundances, respectively (heat maps were obtained by Perseus). Accession of the proteins belonging to the main five clusters are reported.
