# Supplementary material for: Surface Layer Protein Pattern of Levilactobacillus brevis Strains Investigated by Proteomics
Source: Nutrients. 2022 Sep 6;14(18):3679. doi: 10.3390/nu14183679 (PMC9504196; doi:10.3390/nu14183679)
Supplement: Supplementary file 1 [file nutrients-14-03679-s001.zip › Table S1 S2 S3.pdf]

Table S1: DNA extraction, primer and amplification conditions for RAPD-PCR, cluster analysis of band profiles

**DNA extraction**	An aliquot of 2 mL of each overnight culture was centrifuged at 14000 g for 10 min at 4°C and the cell pellet was subjected to DNA extraction according to Querol et al. [30], with the addition of lysozyme (25 mg/mL, Merck, Darmstadt, Germany) and mutanolysin (10 U/mL, Merck) for bacterial cell-wall digestion. Quantity and purity of the DNA were assessed by optical reading at 260 and 280 nm.
**Primer and Amplification conditions**	M13: 5'GAGGGTGG CGGTTCT 3' (Huey & Hall, 1989)[31]. Thirty-five cycles of: 94°C for 1 min, 40°C for 20 s, ramp to 72°C at 0.5°C/s, 72°C for 2 min;<br><br>D8635: 5'GAGCGGCCAA AGGGAGCAGAC 3' (Akopyanz et al., 1992)[32], . Thirty cycles of 94°C for 1 min, 42°C for 1min, 72°C for 90 s, 72°C for 10 min.<br><br>The amplification reactions were performed by a Mastercycler gradient (Eppendorf, Hamburg, Germany) in a 25 μL reaction volume containing 10 mmol/L Tris-HCl (pH 8.3), 50 mmol/L KCl, 200 μmol/L of each dATP, dGTP, dCTP and dTTP, 1.5 mmol/L MgCl2, 1 μ mol/L primer, 80 ng DNA and 1.25 U Taq-DNA polymerase (Thermo Fisher Scientific).
**Electrophoresis conditions**	The amplification products were separated by electrophoresis on 1.5% (w/v) agarose gel (Merck) in 0.5 TBE buffer and then stained for 20 min in 1xTBE containing 1x gelRed (Biotium, Inc. Fremont, CA, USA).
**Agarose gel acquisition**	Agarose gels were digitally captured by the GEL DOC XR System (Bio-Rad, Hercules, CA, USA) using the software Quantity One Analysis (Bio-Rad) and analyzed with the pattern analysis software package, Gel Compare II Version 6.6 (Applied Maths, Sint-Martens-Latem, Belgium).
**Calculation of similarities**	Calculation of similarities in the profiles of bands was based on the Pearson product-moment correlation coefficient. Dendrograms were obtained by means of the Unweighted Pair Group Method using Arithmetic Average (UPGMA) clustering algorithm. Strains were clustered using an 85% homology cut-off, above which strains were considered to be closely related and assigned to the same cluster.

Akopyanz, N; Bukanov, N.O.; Westblom, T.U.; Kresovich, S.; Berg, D.E. DNA diversity among clinical isolates of *Helicobacter pylori* detected by PCR based RAPD fingerprinting. Nucleic Acids Res **1992**, 20, 5137–5142. https://doi.org/10.1093/nar/20.19.5137

Huey, B.; Hall, J. Hypervariable DNA fingerprinting in *Escherichia coli*. Minisatellite probe from bacteriophage M13. J Bacteriol. **1989**, 171, 2528–2532. https://doi.org/10.1128/jb.171.5.2528-2532.1989.

Table S2: Detailed protocols for extraction and digestion of surface proteins

**Protein extraction**	Bacterial pellets (about 1 g) were suspended in 10 mL of 20% sucrose in 5 mol/L LiCl and incubated for 1 h at 4°C. The samples were centrifuged (7000 g for 15 min at 4°C), and the supernatants were filtered through 0.22 μm nitrocellulose membranes (Merck), Surface protein extracts were precipitated with −20°C pre-chilled pure ethanol (1:9 v/v) by incubating for 2 h at -20°C. The protein pellets were recovered by centrifugation (15000 g for 15 min at 4°C), washed twice with 1 mL of 70% ethanol and solubilized in 0.1 mol/L Tris-HCL, 8 mol/L urea, pH 8.5. Protein concentration was measured by Bradford assay (BioRad).
**Protein tryptic digestion**	Protein samples (20 μg) were diluted to 2 mol/L urea by the addition of 0.1 mol/L Tris-HCl, pH 8.5 and digested with 0.4 μg of Sequencing Grade Modified Trypsin (1:50 w/w) (Promega, Madison, WI, USA). The digestion was carried out at 37°C for 18 h. The obtained peptide mixtures were desalted by solid phase extraction using C18 ZipTip Columns (Merck), conditioned with acetonitrile (ACN) and rinsed with 0.1% formic acid (FA); peptides were loaded in 0.1% FA, eluted with 70% ACN in 0.1% FA, dried in a Speed-Vac centrifuge (Savant) and solubilized in 0.1% FA.

Table S3: Detailed protocols for LC-MS/MS, Protein Identification and Label-Free Quantification (LFQ) Analyses

**LC-MS/MS Analysis**	Peptide mixtures were concentrated and desalted on a trapping pre-column (Acclaim™ PepMap™ 100 C18 HPLC Columns, 0.1 mm × 20 mm, 5 µm, 100 Å, Thermo Fisher Scientific), using 2% ACN in 0.05% FA at a flow rate of 10 µL/min.<br>The peptide separation was performed at 40°C using a C18 column (EASY-Spray™ HPLC Columns, 75 µm × 250 mm, 2 µm, 100 Å, Thermo Fisher Scientific), using 0.1% FA as eluent A and 80% ACN in 0.08% FA as eluent B at a flow rate of 0.3 µL/min and a linear gradient from 2 to 50% B over 60 min, hold for 10 min, from 50 to 90% B over 1 min, hold for 10 min before column re-equilibration to 2% B.<br>Mass spectra were acquired in the m/z range 350-1600. Data acquisition was performed in a data dependent mode Full MS/ddMS2, enabling the acquisition of MS/MS spectra for the ten most intense precursor ions (top ten) and dynamic exclusion of 10 sec. Resolution was set to 70000 for MS spectra acquisition and 17500 for MS/MS spectra acquisition.
**Protein identification**	The MaxQuant software (version 1.6.3.4) was used for processing the MS raw files using the Andromeda search engine. The parameters used for the data-base searches were the following: *Levilactobacillus brevis* (basonym *Lactobacillus brevis*) database (taxon 1580, downloaded from UniProtKB on October 2021, https://www.uniprot.org/) and a contaminant protein data-base (provided by the manufacturer), trypsin as proteolytic enzyme, up to two missed cleavages, oxidation of methionine residues as dynamic modification, 20 ppm mass tolerance for precursor ions and 0.02 Da mass tolerance for MS/MS fragments. The false discovery rate (FDR) was set to 1% at both the protein and peptide levels. The MaxQuant LFQ algorithm was used for protein quantification, using Razor plus Unique Peptides option.
**Label-Free Quantification (LFQ) Analysis**	The Perseus software (version 1.6.0.7) was used for processing the MaxQuant data. Contaminants and reverse hits were removed from the dataset. After a log_2-transformation of the LFQ data, only proteins identified by means of a number of Razor plus Unique Peptides ≥2 in two technical replicates for each biological replicate of at least one strain were considered reliably identified. Proteins selected with these criteria in one strain will be considered specifically expressed by that strain.<br>The imputation of missing values was performed by selecting a downshift of 1.8 and a width of 0.3 standard deviations in a Gaussian distribution of random numbers.<br><br>Perseus – Volcano plot option was applied to calculate statistically significant differences between LFQ values of proteins in A4, PA6, A7 and M4 and those of TS (selected as reference group). A p-value < 0.05 (-Log Student's T-test p-value >1.3) was considered statistically significant. Protein fold changes were calculated as the difference of log2 of mean protein LFQ values in A4, PA6, A7, M4 and log2 of mean protein LFQ in TS. Proteins with a fold change >1.5 were selected as more abundant and < -1.5 as less abundant.<br><br>The putative relative abundance level of each Slp in a single strain was calculated using a spectral counting approach and inferred by the MS/MS

counts (i.e. MS/MS spectra attributed to Razor and Unique Peptides for each Slp). The normalized spectral abundance factor (NSAF) for each Slp was calculated as the number of MS/MS counts divided by the protein length (thus obtaining the SAF value) and normalized to the sum of SAFs in each sample. The relative abundance level of each Slp in a strain was calculated as the ratio between the NSAF of each Slp and the sum of NSAF for all the Slps in that strain.
